# Factors Influencing Herpes Zoster Vaccine Utilization Among Adults Aged 50 and Above Attending Primary Healthcare Center in Saudi Arabia: A Cross-Sectional Study

**DOI:** 10.7759/cureus.66761

**Published:** 2024-08-13

**Authors:** Mohammed Almakhdob, Mohie Selim, Abuobieda Abdalrouf

**Affiliations:** 1 Preventive Medicine, Family and Community Medicine Administration, Prince Sultan Military Medical City (PSMMC), Riyadh, SAU

**Keywords:** cross-sectional study, saudi arabia, older adults, vaccine utilization, varicella-zoster virus, shingles, herpes zoster

## Abstract

Introduction: Herpes zoster, or shingles, is a significant health concern for older adults caused by the varicella-zoster virus (VZV) reactivation. The availability of effective herpes zoster vaccines offers a crucial preventive measure to reduce the incidence and severity of this condition. However, the uptake of the available vaccine remains suboptimal, especially among adults aged 50 and above. Understanding the factors that influence vaccine utilization is not only essential for developing strategies to improve vaccination rates but also has the potential to significantly reduce the disease burden.

Methods: This cross-sectional study aimed to identify factors influencing herpes zoster vaccine utilization among adults aged 50 and above attending primary healthcare center in Saudi Arabia. Data were collected using a validated questionnaire administered to visitors attending Al-Wazarat Primary Healthcare Center in Riyadh. Descriptive and inferential statistics were employed to analyze the data.

Results: A total of 403 participants were included in the study, with 73.7% of participants having heard of the disease, yet only 34.2% had received the vaccine. Vaccine uptake was significantly associated with gender, educational level, and healthcare provider recommendations. Common barriers to vaccination included fear of side effects, lack of perceived susceptibility, concerns about vaccine effectiveness, and access to healthcare facilities.

Conclusion: Herpes zoster vaccine utilization among older adults in Saudi Arabia is relatively low despite moderate awareness. Enhancing public education through targeted campaigns and strengthening healthcare provider recommendations are crucial to improving vaccine uptake. Addressing specific barriers and misconceptions is essential to reduce the burden of herpes zoster in this population. The need for future research to explore strategies to overcome these challenges and promote higher vaccination rates is urgent and important.

## Introduction

Herpes zoster, commonly known as shingles, is caused by the reactivation of the varicella-zoster virus (VZV), the same virus responsible for chickenpox, which is characterized by a painful, unilateral vesicular rash. Herpes zoster typically affects older adults and immunocompromised individuals, causing significant morbidity and a decreased quality of life [1-4].

The epidemiology of herpes zoster reveals a significant burden, especially among older adults. Globally, the incidence of herpes zoster ranges from three to five per 1,000 person-years in the general population and increases dramatically to eight to 12 per 1,000 person-years in those aged 50 and above [5-7]. In Saudi Arabia, similar trends have been observed with increasing incidences among the elderly population, correlating with age-related decline in cell-mediated immunity [8]. Complications such as postherpetic neuralgia, a chronic pain condition, and herpes zoster ophthalmicus can persist long after the rash resolves, contributing further to the disease burden [9-11].

The prevention of herpes zoster has become a pivotal strategy for mitigating its impact. The advent of vaccines has revolutionized prevention approaches. Historically, the live attenuated zoster vaccine (ZVL) was introduced to reduce the incidence of herpes zoster and postherpetic neuralgia [12,13]. More recently, a recombinant adjuvanted zoster vaccine (RZV) has shown superior efficacy and is now preferred in many national immunization programs [14]. The RZV has demonstrated over 90% efficacy in preventing herpes zoster across various age groups, including those aged 50 and above [15,16].

The vaccine history for herpes zoster began with the ZVL, which was recommended primarily for those aged 60 and above [12]. Despite its effectiveness, its uptake was hindered by contraindications in immunocompromised individuals and decreased efficacy with advancing age [17,18]. Additionally, real-world assessments have shown that vaccine effectiveness wanes over time, from 69% (95% CI 65%-74%) in the first year after vaccination to 45% (95% CI 29%-57%) by the third year [19]. The introduction of the RZV addressed several of these limitations, offering enhanced protection and an improved safety profile [17,18]. Studies have shown that the RZV maintains high efficacy for at least four years following vaccination, with ongoing studies likely to provide further insights into long-term protection [20]. The significant adverse effects associated with the RZV are localized injection site reactions, such as pain and redness, and systemic reactions, including fatigue and fever. These reactions are generally mild to moderate in intensity and transient [21].

Despite the availability of effective vaccines, several factors influence vaccine utilization, particularly in Saudi Arabia. Socioeconomic status, educational background, access to healthcare, and cultural beliefs significantly impact vaccine uptake [22]. In Saudi Arabia, awareness about herpes zoster among the older population is relatively moderate and the benefits of vaccination remain relatively low among the general populace [22,23]. Healthcare providers in primary healthcare centers (PHCs) play a crucial role in educating and encouraging vaccination, yet their recommendations are only sometimes heeded. Additional barriers include misconceptions about vaccine safety and efficacy, fear of adverse effects, and logistical challenges such as availability and cost [24].

Addressing the issue of low vaccine utilization requires a multifaceted approach. Enhancing public awareness through targeted educational campaigns emphasizing the benefits and safety of the RZV is essential. Training healthcare providers to communicate the importance of vaccination and provide personalized recommendations can also improve vaccine uptake. Additionally, integrating the herpes zoster vaccine into routine immunization schedules for adults and ensuring availability and affordability can help bridge the gap. Managing the gap in vaccine utilization involves understanding and addressing the specific barriers faced by the Saudi Arabian population.

This study aims to determine the percentage of individuals who have completed the recommended herpes zoster vaccine dose and recognize factors influencing vaccine acceptance. In addition, we strive to identify the rate of persons aged 50 and above who previously developed herpes zoster and project the proportion of individuals who have not received any vaccine doses. These objectives aim to provide a comprehensive understanding of herpes zoster vaccine utilization and barriers among older adults in Saudi Arabia.

## Materials and methods

Study setting and population

This single-center study was conducted at Al-Wazarat PHC Center in Prince Sultan Military Medical City (PSMMC) in Riyadh, Saudi Arabia. This setting was selected due to its accessibility and the diverse patient population it serves, which provided a comprehensive sample for the study. 

The study population comprised adults aged 50 and above who attended Alwezarat PHC Center from May to July 2024. This age range was selected based on guidelines recommending immunization against herpes zoster for individuals in this demographic, who are at higher risk for the disease and its complications.

Inclusion criteria

Inclusion criteria included Saudi citizens or residents aged 50 years and older living in Saudi Arabia. Visitors to Saudi Arabia were excluded to ensure that the study population was representative of long-term residents and citizens who were more likely to access ongoing healthcare services in the country.

Sample size and technique

The sample size for this study was 403 participants. This number was calculated using the WHO sample size calculator to ensure adequate statistical power and representativeness. A convenience sampling technique was employed to select participants. This approach was chosen for its practicality and efficiency in recruiting sufficient participants within the study period.

Data collection

The data collection tool was a questionnaire constructed by drawing questions from similar studies in the literature. The questionnaire was validated through reviews by two experts to ensure its validity and reliability.

The internal consistency of the questionnaire assessing factors influencing Herpes Zoster Vaccine utilization among adults aged 50 and above was evaluated using Cronbach's alpha. The questionnaire items covered various domains, including demographic information, knowledge about herpes zoster and the vaccine, perceptions and attitudes toward the vaccine, vaccine utilization, and barriers to vaccination. The overall Cronbach's alpha was 0.838, indicating good internal consistency across all items.

The questionnaire consisted of close-ended and multiple-choice questions divided into following three sections. Section A - sociodemographic profile, comprising six questions on age, gender, education, employment status, and health status. Section B - ten questions regarding knowledge and attitudes about herpes zoster. Section C - six questions with multiple parts about vaccination status and practices regarding the herpes zoster vaccine.

Data were collected via a self-administered questionnaire. For participants who could not read or write, an assistant team was available to help them complete the self-administered questionnaire. The questionnaire underwent a pilot study with 20 participants to ensure clarity and reliability, and it was refined based on feedback from this pilot study.

Data analysis

The collected data were analyzed using the IBM Statistical Package for Social Sciences (IBM Corp., Armonk, NY) version 25. Descriptive statistics were used to summarize the sociodemographic characteristics and responses to the questionnaire. Inferential statistics were applied to assess differences and associations between variables. The Chi-square test for independence was used to determine if there was a statistically significant association between categorical variables. Logistic regression analysis was used to model the probability of vaccination status based on several predictor variables. A p-value of less than 0.05 will be considered statistically significant.

Ethical considerations

The study sought approval from the Institutional Ethics Committee Board of PSMMC. Participants were informed about the study's objectives, and written consent was obtained from all participants. They were assured that their participation was voluntary, and they could withdraw from the study at any time without any consequences. Participants' information was confidential, and the data collected were used solely.

## Results

Demographic characteristics

The study population consisted of 403 adults aged 50 and above attending a PHC in Saudi Arabia. The mean age of the participants was 60.38 years (SD = 6.774). Of the participants, 47.4% were male (mean: 61.26 years (SE: 0.482)) and 52.6% were female (mean: 59.59 years (SE: 0.467)). Most participants were married (63.3%), while 14.6% were divorced, 12.9% were widowed, and 9.2% were single. Regarding occupational status, 37.0% were retired, 31.8% were housewives, 20.6% were employees, 7.4% were without work, and 3.2% were military personnel. Most participants had an educational level of intermediate (31.5%) or primary (31.3%), while 17.1% had a high school education, 15.1% had no qualification, and 5.0% had a university education. A significant portion of the sample (79.4%) had multiple comorbidities, 19.6% had a single comorbidity, and only 1.0% had no comorbidities (Table 1).

**Table 1:** Distribution of the demographic characteristics of the participants attending PHC in Riyadh, Saudi Arabia. PHC - Primary healthcare center

Variable	Category	Count	Percent (%)	Mean age (years)
Age group	50 to 59 years	182	45.2	54.40
	60 to 69 years	195	48.4	64.58
	70 to 79 years	20	5.0	72.45
	80 to 90 years	3	0.7	84.67
Gender	Male	191	47.4	61.26
	Female	212	52.6	59.59
Marital status	Single	37	9.2	54.41
	Married	255	63.3	61.20
	Divorced	59	14.6	58.46
	Widow	52	12.9	62.81
Occupational status	Employee	83	20.6	55.40
	Retired	149	37.0	62.67
	Military	13	3.2	54.54
	Without work	30	7.4	59.93
	Housewife	128	31.8	61.65
Educational level	No qualification	61	15.1	64.21
	Primary	126	31.3	60.79
	Intermediate	127	31.5	61.38
	High school	69	17.1	55.35
	University	20	5.0	57.15
Comorbidities: DM HTN CVD hypothyroidism	No comorbidities	4	1.0	54.50
	Single comorbidity	79	19.6	57.75
	Multiple comorbidities	320	79.4	61.11
Source of information	No source of information	124	30.8	61.35
	Single source of information	162	40.2	60.08
	Multiple sources of information	117	29.0	59.78
Type of source of information	Physician	124	30.8	60.23
	Social media	72	17.9	58.00
	TV	71	17.6	60.93
	Friends and acquaintances	66	16.4	58.58
	Health awareness programs	53	13.2	59.85

Vaccine utilization, awareness, and barriers to vaccination

In terms of vaccine utilization, 34.2% of participants had taken the herpes zoster vaccine, and 20.6% had completed the recommended two doses. Among those aware of the vaccine, 37.5% believed it was effective, while 57.6% were uncertain about its effectiveness. Additionally, 67.0% knew the vaccine was available free to individuals above 50 years old in Saudi Arabia (Table 2).

**Table 2:** Different vaccine-related variables including knowledge, awareness, attitudes, and vaccine utilization.

Variable	Yes (Percent)	No (Percent)	I do not know (Percent)
Vaccine Utilization			
Have you taken herpes zoster vaccine?	138 (34.2)	265 (65.8)	N.A.
If you take only one dose, do you intend to take the second? (55)	47 (85.5)	8 (14.5)	N.A.
If your doctor recommends herpes zoster vaccine, will you get vaccinated?	269 (66.7)	134 (33.3)	N.A.
How many doses of herpes zoster have you taken so far?	N.A.	N.A.	N.A.
One dose	55 (13.6)	N.A.	N.A.
2 doses (completed)	83 (20.6)	N.A.	N.A.
No doses	265 (65.8)	N.A.	N.A.
Health-related variables			
Have you had chickenpox in the past?	303 (75.2)	57 (14.1)	43 (10.7)
Have you ever been had herpes zoster?	17 (4.2)	386 (95.8)	N.A.
Knowledge and awareness			
Have you heard of herpes zoster disease?	297 (73.7)	106 (26.3)	N.A.
Do you know the disease symptoms?	165 (40.9)	238 (59.1)	N.A.
Would you look for more information from your doctor about the herpes zoster vaccine?	246 (61.0)	153 (38.0)	4 (1)
Perceptions and attitudes			
Do you know about herpes zoster vaccine?	278 (69.0)	125 (31.0)	N.A.
Do you think the vaccine is effective to prevent the disease?	151 (37.5)	20 (5.0)	232 (57.6)
Do you know that the vaccine is available for free to individuals above 50 years old in KSA?	270 (67.0)	133 (33.0)	N.A.

Among those who had taken only one dose, 85.5% planned to complete the vaccine course. When participants were asked if they would get vaccinated if recommended by their doctor, 66.7% indicated they would, whereas 33.3% indicated they would not. Of the 33.3% who indicated refusal of doctors’ recommendation, the main barriers to vaccination included fear of side effects (37.3%), problems accessing healthcare facilities (27.6%), a general opposition to vaccines (17.2%), a lack of perceived information on severity and length of the disease (13.4%), concerns about vaccine effectiveness (11.2%), and a lack of perceived susceptibility to the disease (4.5%) (Table 3).

**Table 3:** Barriers to vaccination reported by participants who showed unwillingness to accept physicians’ recommendations to vaccination.

Barriers to vaccination (134)	Yes (Percent)	No (Percent)
I am afraid of the side effects of vaccine	50 (37.3)	84 (62.7)
I have a problem going to the doctor to get the vaccine	37 (27.6)	97 (72.4)
I am against vaccines in general	23 (17.2)	111 (82.8)
I don’t think the disease is harmful or lasts for long time	18 (13.4)	116 (86.6)
I think vaccination isn't quite as effective	15 (11.2)	119 (88.8)
I don't think I’m highly susceptible to the disease	6 (4.5)	128 (95.5)

Regarding vaccine awareness, 73.7% of participants had heard of herpes zoster disease, 40.9% knew the symptoms, and 69.0% were aware of the herpes zoster vaccine. The most common sources of information for participants were physicians (30.8%), followed by social media (17.9%), TV (17.6%), friends and acquaintances (16.4%), and health awareness programs (13.2%), respectively (Table 1). The study found that 4.2% of participants had previously developed herpes zoster. The proportion of individuals who had not received any doses of the herpes zoster vaccine was 65.8% (Table 2).

Factors influencing vaccine uptake

Chi-square tests revealed that gender (χ² = 10.750, p = 0.001), occupational status (χ² = 9.968, p = 0.041), and educational level (χ² = 12.739, p = 0.013) were significantly associated with herpes zoster vaccine uptake. Specifically, males were more likely to have taken the vaccine than females, and those who are retired and with “University” educational levels were more likely to have taken the vaccine compared to others (Table 4).

**Table 4:** Chi-square test between Shingrix vaccine utilization and demographic variables among participants attending PHC in Riyadh, Saudi Arabia. PHC - Primary healthcare center

Variable	Category	Vaccine status				
		Yes	No	Total	Chi-square value	P-value
Gender	Male	81	110	191	10.750	0.001
	Female	57	155	212		
Marital status	Single	13	24	37	5.656	0.130
	Married	97	158	255		
	Divorced	15	44	59		
	Widow	13	39	52		
Occupational status	Employee	32	51	83	9.968	0.041
	Retired	62	87	149		
	Military	4	9	13		
	Without work	8	22	30		
	Housewife	32	96	128		
Educational level	No qualification	12	49	61	12.739	0.013
	Primary	45	81	126		
	Intermediate	40	87	127		
	High school	30	39	69		
	University	11	9	20		
Number of comorbidities	No comorbidities	2	2	4	0.706	0.703
	Single comorbidity	25	54	79		
	Multiple comorbidities	111	209	320		

Logistic regression analysis

A logistic regression analysis identified significant predictors of herpes zoster vaccine uptake. The model explains approximately 30.3% of the variance in vaccination status, indicating a moderate level of predictive power, after considering multiple predictor variables simultaneously and assessing the effect of each variable on the outcome (vaccination status), controlling for the influence of other variables in the model (Table 5). The model correctly classified 71.5% of the cases, with higher accuracy in predicting those who did not get vaccinated (84.9%) compared to those who did (45.7%).

**Table 5:** Logistic regression model for Shingrix vaccine utilization among participants attending PHC in Riyadh, Saudi Arabia. PHC - Primary healthcare center

Variable	B	S.E.	Wald	df	Sig.	Exp(B)
Age	0.004	0.020	0.032	1	0.858	1.004
Gender	-0.784	0.288	7.432	1	0.006	0.457
Marital status	0.051	0.167	0.093	1	0.760	1.052
Occupational status	0.006	0.098	0.004	1	0.950	1.006
Educational level	0.176	0.132	1.770	1	0.183	1.192
Number of comorbidities	0.743	0.295	6.331	1	0.012	2.102
Source of information	1.380	0.184	56.182	1	0.000	3.975
History of chickenpox	-0.284	0.210	1.830	1	0.176	0.753
History of herpes zoster	-0.911	0.589	2.389	1	0.122	0.402
Constant	-1.004	1.994	0.254	1	0.614	0.366

Gender

The odds of males receiving the vaccine are 54.3% lower than females (OR = 0.457, p < 0.01), and this contradicts the results of the Chi-square test. This means that, after adjusting for other factors in the model (e.g., age, marital status, educational level, number of comorbidities, source of information, history of chickenpox, history of herpes zoster), males were less likely to receive the vaccine compared to females. 

Number of Comorbidities

Each additional comorbidity increases the odds of vaccination by 2.1 times (OR = 2.102, p < 0.02).

Source of Information

Those who receive their information from specific sources are almost four times more likely to get vaccinated (OR = 3.975, p < 0.001).

## Discussion

The findings from our study reveal essential insights into the utilization of Herpes Zoster vaccines among adults aged 50 and above attending Al-Wazarat PHC in Riyadh, Saudi Arabia. Our analysis identified that only 20.6% of the participants had completed the recommended two herpes zoster vaccine doses. Among those who had taken only one dose (55 participants (13.6%)), 85.5% planned to complete the vaccine course. This low completion rate is consistent with findings from previous studies in Saudi Arabia. For instance, Almuammar et al. found that only 7.7% of their participants had received the herpes zoster vaccine despite 57.2% having heard about it [23]. Similarly, Bohamad et al. reported an 8% vaccination rate, highlighting the significant gap between awareness and vaccine uptake [22]. Moreover, the completion rates of the herpes zoster vaccine doses in our study varied significantly across different age groups. Participants aged 50-59 had the highest completion rates (22.5%), followed by those aged 60-69 (20.5%), while participants aged 70-79 and 80-90 had significantly lower rates. This trend is inconsistent with findings from the UK, where vaccine uptake increases with age [25]. In our study, the awareness of the herpes zoster disease and vaccine was moderate, with 73.7% of participants having heard of the disease, 40.9% knew the symptoms, and 69.0% were aware of the herpes zoster vaccine. These findings are similar to those reported by Alhothali et al. in Saudi Arabia (83.2% of participants had heard of the disease and 55.8% were aware of the herpes zoster vaccine) and Draper in the USA (99% % of participants were aware of the disease) [26,27]. On the other hand, some studies reported moderate awareness of the disease and relatively low awareness of the presence of the vaccine such as Al-Khalidi et al. in the United Arab Emirates who reported that 64.3% of participants were aware of herpes zoster and 14.8% of participants were aware of the Herpes Zoster vaccine and Del Signore et al. in France who reported 87.6% of participants had heard of the disease and 11.9% were aware of the herpes zoster vaccine [28,29].

The awareness about the disease increased in Saudi Arabia due to national awareness programs from the Ministry of Health on TV, radio, and social media, in addition to disease-related campaigns introduced to the population [30]. These awareness programs and campaigns should increase and address different sectors of the population according to their educational level and culture, as 232 (57.6%) of participants in our study lack information on the effectiveness of vaccines in preventing the disease.

The discrepancy between awareness and uptake underscores a critical issue in public health: awareness alone does not necessarily lead to higher vaccination rates. This gap suggests that other factors, such as access to the vaccine, perceived necessity, and healthcare provider recommendations, play substantial roles in influencing vaccination behavior.

Factors influencing vaccine acceptance

Our study aimed to recognize the factors influencing vaccine acceptance positively and negatively. We found that participants with a “University” educational level were more likely to accept the vaccine. Conversely, Almuammar et al. found that participants with primary education were more likely to accept the vaccine than those with higher education. This finding contradicts the general assumption that education correlates with better health practices, suggesting that educational campaigns need to be tailored differently for various educational backgrounds [23]. 

Gender differences also emerged as significant, with men being more likely to accept the vaccine than women, a finding corroborated by Alhothali et al. [26]. This highlights the necessity for gender-specific approaches in health communication strategies to improve vaccine uptake among women. However, in our logistic regression model, after adjusting for other factors in the model (e.g., age, marital status, educational level, number of comorbidities, source of information, history of chickenpox, history of herpes zoster), males were more likely to receive the vaccine compared to females (Table 5).

Willingness to complete the vaccine course

A promising finding from our study is that 85.5% of participants who had taken the first dose (55 participants) planned to complete the vaccine course, which indicates a potential for improving completion rates through targeted interventions. The literature suggests that healthcare provider recommendations significantly boost willingness to complete vaccination courses. Al-Orini et al. demonstrated that physician advice could increase acceptance rates from 25% to 74.2% among patients [31]. Therefore, integrating more robust healthcare provider engagement and follow-up could enhance vaccine completion rates.

Past history of disease incidence and vaccine uptake

Our secondary objective was to identify the rate of previous herpes zoster disease among participants, which stood at 4.2%, which aligns with the low incidence of the disease reported by Alhumaid et al. (4%) among the study participants [30]. 

Non-vaccinated individuals

A significant proportion of our study population (65.8%) had not received any doses of the herpes zoster vaccine, which aligns with other studies, such as Al-Khalidi et al., who found that 96.7% of participants had not taken the vaccine despite a general willingness to do so if recommended by healthcare professionals [28]. The lower vaccine uptake or lack of willingness to join the vaccination program, especially in older age groups, may be due to various factors such as limited access to healthcare services, higher prevalence of contraindications, or a lower perceived need for vaccination among the elderly. These findings highlight the need for targeted interventions to improve vaccine uptake, especially among older individuals who are at higher risk of complications from herpes zoster. The main barriers identified include lack of awareness, safety concerns, and perceived lack of risk. These findings indicate that comprehensive educational campaigns and strong healthcare provider endorsements are crucial to improving vaccination rates.

Implications for public health and future research

Our study's findings have several implications for public health strategies. First, enhancing awareness campaigns to inform, persuade, and facilitate action is essential. Educational interventions should be tailored to address specific barriers, such as safety concerns and perceived necessity, and delivered in a culturally sensitive manner. 

Moreover, healthcare providers play a pivotal role in influencing vaccine uptake. Training programs that equip healthcare providers with the skills to effectively communicate the benefits and safety of the herpes zoster vaccine could significantly impact vaccination rates. Al-Shanbari et al. highlighted that a healthcare provider's recommendation is one of the most influential factors in vaccine acceptance [8].

Future research should explore the reasons for the educational and gender disparities in vaccine acceptance. Qualitative studies could provide deeper insights into personal beliefs and cultural factors influencing vaccine hesitancy. Additionally, longitudinal studies tracking vaccine uptake and completion over time help evaluate the long-term effectiveness of implemented interventions.

Study limitations

Despite valuable insights, our study has several limitations. The sample size of 403 participants from a single PHC, which may not capture regional variations in vaccine acceptance and healthcare access, may limit the generalizability of our findings to the broader population of older adults in Saudi Arabia. The cross-sectional design restricts our ability to establish causality between identified factors and vaccine uptake. Self-reported data may be subject to recall bias, especially regarding past vaccine behavior and disease history. Potential confounding variables, such as underlying health conditions and socioeconomic status, may also influence the results but must be comprehensively controlled for in our analysis. These limitations suggest more extensive, multi-center longitudinal studies to validate our findings and provide a more comprehensive understanding of the factors affecting herpes zoster vaccine utilization in this population.

## Conclusions

In conclusion, while awareness of the herpes zoster vaccine among adults aged 50 and above in Saudi Arabia is moderate, vaccine uptake and completion rates remain relatively low. Educational background, gender, and healthcare provider recommendations significantly influence vaccine acceptance. Addressing the barriers to vaccination through tailored educational campaigns and more robust healthcare provider engagement is essential. Future research should focus on understanding the factors of vaccine hesitancy and evaluating the effectiveness of targeted interventions.

## Appendices

**Table 6:** Questionnaire for herpes zoster vaccination and factors affecting vaccine utilization among adults over 50 in the Kingdom of Saudi Arabia.

Section A: social and demographic details		
Name (Optional)		
Age		
Gender Male Female		
Marital status	Single - Married Divorced - Widow	
Occupational status	Employee - Retired - Military - Without work Housewife	
Educational level	No Qualification Primary - Intermediate - High School University	
Do you suffer from any of these chronic diseases? (you can choose more than one)	Hypertension	Hypothyroidism
	Diabetes	Renal Diseases
	Hyperlipidemia	Rheumatoid arthritis
	Cardiology diseases	Depression
	Asthma	Others: ………..
Section B: knowledge and trends about herpes zoster		
Have you had chickenpox in the past?	Yes - No - I don't remember	
Have you heard of herpes zoster disease?	Yes - No	
Have you ever been had of herpes zoster?	Yes - No	
To your knowledge, what are the possible symptoms of herpes zoster	Pain - rash. - Eye Problems - Others: …….. ­ I don't know.	
To your knowledge, is the pain associated with the herpes zoster?	Mild Intermediate - Severe - I don't know	
Does the chronic pain associated with the herpes zoster affect normal daily activities	Slightly Considerably Extremely I don’t know	
Do you know about herpes zoster vaccine?	Yes - No	
What is your source of information about the herpes zoster vaccine? (you can choose more than one)	Doctor Friends/Acquaintances Government Organization Health awareness programs Internet Radio Family TV Others: Specify……………..	
Do you think the vaccine is effective to prevent the disease?	Yes - No - I don't know	
Who do you think is most susceptible to herpes zoster?	Adults under the age of 40 - People of 41 to 49 years old - Adults over 50 years old - I don't know	
Section C: herpes zoster vaccine		
Do you know that the vaccine is available for free to individuals above 50 years old in KSA?	Yes - No	
Have you taken herpes zoster vaccine?	Yes - No	
How many doses of herpes zoster have you taken so far?	One Dose - 2 doses (completed). – No doses If you take only one dose, do you intend to take the second? Yes - No	
If your doctor recommends herpes zoster vaccine, will you get vaccinated?	Yes - No -	
If you have answered the previous question with no, why? (you can choose more than one)	I don't think I’m highly susceptible to the disease I think vaccination isn't quite as effective I have a problem going to the doctor to get the vaccine I am afraid of the side effects of vaccine Iam against vaccines in general I don’t think the disease is harmful or lasts for long time Others (Please specify) _____________________	
Would you look for more information from your doctor about the herpes zoster vaccine?	Yes - No - I don’t know	
